# The impacts of global atmospheric circulations on the water supply in select watersheds in the Indonesian Maritime Continent using SPI

**DOI:** 10.1016/j.heliyon.2023.e15604

**Published:** 2023-04-19

**Authors:** Fauzan Ikhlas Wira Rohmat, Wendi Harjupa, Dede Rohmat, Faizal Immaddudin Wira Rohmat

**Affiliations:** a Water Resources Development Center, Bandung Institute of Technology (ITB), CIBE Building 5th Floor, Jalan Ganesa No. 10, Bandung, 40132, West Java, Indonesia; b Research Center for Climate and Atmosphere, National Research and Innovation Agency (BRIN), Bandung, 40173, West Java, Indonesia; c Department of Geography, Indonesia University of Education (UPI), Jalan Dr. Setiabudhi No. 229, Bandung, 40154, West Java, Indonesia; d Faculty of Civil and Environmental Engineering Bandung Institute of Technology (ITB), FTSL Building, Jalan Ganesa No. 10, Bandung, 40132, West Java, Indonesia

**Keywords:** Atmospheric circulations, Standardized precipitation index, Streamflow, Watersheds

## Abstract

Despite the diverse atmospheric circulations affecting the Indonesian Maritime Continent (IMC), i.e., El Nino Southern Oscillation-ENSO, Indian Ocean Dipole-IOD, Madden Julian Oscillation-MJO, Monsoon, there is a lack of research on their interaction with hydrological events in watersheds. This study fills this gap by providing insights into the dominant atmospheric events and their correlation with the water supply in three characteristic watersheds, i.e., Tondano (north/Pacific Ocean), Jangka (south/Indian Ocean), and Kapuas (equatorial/interior) in IMC. The research used the standardized precipitation index for the 1-monthly (SPI1), 3-monthly (SPI3), and 6-monthly (SPI3) scale generated from 23 years (2000–2022) of monthly historical satellite rainfall data. The analysis compared each location's SPI indices with the monthly Nino 3.4, Dipole Mode Index (DMI), MJO (100E and 120E), Monsoon index, and streamflow data. The result shows that the dominant atmospheric events for the Tondano watershed were ENSO, IOD, and MJO, with correlation values of −0.62, −0.26, and −0.35, respectively. The MJO event was dominant for the Kapuas watershed, with a correlation value of −0.28. ENSO and IOD were dominant for the Jangka watershed, with correlation values of −0.27 and −0.28, respectively. The monsoon correlated less with the SPI3 in all locations, while it modulates the wet and dry period pattern annually. Most intense dry periods in Tondano occur with the activation of El Nino, while the intense wet period occurs even in normal atmospheric conditions. Most intense wet periods in Jangka occur with the activation of La Nina, while the intense dry period occurs even in normal atmospheric conditions. The occurrence of MJO compensates for the intense wet and dry periods in Kapuas. The correlation among SPI3, atmospheric circulation, and streamflow in the diverse watershed characteristics in the IMC watersheds could give strategic information for watershed management and applies to other watersheds with similar atmospheric circulation characteristics.

## Abbreviations

IMC: Indonesian Maritime Continent
ENSO: El Nino Southern Oscillation
IOD: Indian Ocean Dipole
MJO: Madden-Julian Oscillation
NS: Northerly Surge
SS: Southerly Surge
SPI: Standardized Precipitation Index
SPI1: 1-Monthly SPI
SPI3: 3-Monthly SPI
SPI6: 6-Monthly SPI

## 1 Introduction

Indonesian Maritime Continent (IMC) has a unique hydro-meteorological cycle due to its position [1]. Located in the equatorial area and between Asia and Australia, the Monsoon cycle modulates the wet and dry seasons annually as IMC's primary season. This area is also located between the Pacific and the Indian Ocean, directly influenced by the ocean-atmosphere circulation, such as El Nino Southern Oscillation (ENSO) and Indian Ocean Dipole (IOD). Especially for the equatorial region, Madden-Julian Oscillation (MJO) also directly influences IMC's hydro-meteorological cycle [[2], [3], [4], [5], [6]]. Watersheds rely on rainfall for their supply of surface and groundwater. In the hydro-meteorological cycle, the high and low water resource supply depends on the amount of rainfall in some period [[7], [8], [9]]. In normal conditions, the wet season occurs from November to March (NDJFM), which usually coincides with the strengthening of the Asian Monsoon, where the wind circulation blows from the continent of Asia to the IMC region. Meanwhile, the dry season generally starts from April to October (AMJJASO), related to the ongoing Australian Monsoon, where the wind circulation pattern blows from the Australian territory to the Indonesian territory [[10], [11], [12], [13], [14], [15], [16]]. However, this condition is only sometimes typical and consistent due to some ENSO, IOD, and MJO event disturbances.

The occurrence of each ENSO, IOD, and MJO event brings variations to the amount, frequency, and intensity of rainfall. These may result in some abnormal seasons such as severe or long drought, excess water in the rainy season, high rain in the dry season, or long dry in the rainy season. Generally, the western and eastern ocean-atmosphere interactions affect the intensity and duration of the dry and wet seasons. The ENSO and IOD are the two global atmospheric circulations that modulate ocean-atmosphere interaction variability, including IMC's hydrological patterns [[17], [18], [19]]. ENSO has two anomaly conditions – the El Nino and La Nina, while IOD has two anomaly conditions – Positive and Negative IODs. El Nino and Positive IOD are often associated with long dry phenomena and vigorous drought intensity in the IMC region. Meanwhile, the presence of La Nina and Negative IOD is often associated with long rainy season conditions and extreme rains [[20], [21], [22]]. Regarding distribution, rainfalls in IMC are also affected by strong and weak Madden Julian Oscillation (MJO) throughout the year. Previous studies have shown that the MJO can influence the frequency and duration of global precipitation events [4,[23], [24], [25], [26]]. It shows that MJO also has a considerable role in IMC's wet and dry periods.

The anomaly of rainfall causing long dry or wet periods could be identified better with the Standardized Precipitation Index (SPI) – the drought index. The index uses long historical precipitation data [27,28] to determine the occurrence of the dry and wet periods over historical temporal data. It has been studied before that global atmospheric circulation like ENSO, IOD, MJO, and Monsoon also impact the SPI index [[29], [30], [31], [32], [33]]. It also impacts the variation of streamflow and water availability in watersheds [33]. With the historical dry and wet period from SPI, the future occurrence of that period in association with the rainfall anomaly related to atmospheric circulation could be projected.

Although the IMC has a general rainfall pattern correlated with those events [21,34], the specific location may have different dominant atmospheric circulation influences. Differences in dominant atmospheric influences impact differences in the characteristics of water availability in a watershed [33,35,36]. Despite the diverse atmospheric circulations affecting the IMC, there is a lack of research on their interaction with hydrological events in watersheds. This study fills this gap by providing insights into the dominant atmospheric events and their correlation with the water supply in the characteristic watersheds. If the characteristics of each watershed are known, water resources management can be carried out specifically to have a more effective impact. Until now, the water resources management in Indonesia has only considered seasonal changes that the Monsoon influences and has not considered the effects of other atmospheric circulation, which tend to generate abnormal conditions.

This study focuses on three watersheds with very different geographic locations: the Kapuas, Tondano, and Jangka. Those three watersheds represent IMC's north/Pacific Ocean, south/Indian Ocean, and equatorial/interior areas, respectively. This study examines the changes in rainfall patterns in dry and wet periods and determines what atmospheric circulation is dominant for these changes. Then, it will be determined how strong the relationship between the dry and wet periods is to water availability in each watershed. This study would lead to an understanding of how water availability in IMC relates to the surrounding hydro-meteorological phenomena. Knowing the dominant atmospheric circulation of water availability in a watershed, we could bring the water resources management into more specific, effective, and efficient measures according to the respective watershed characteristics.

## 2 Materials and methods

### 2.1 Study domain

**Fig. 1:**
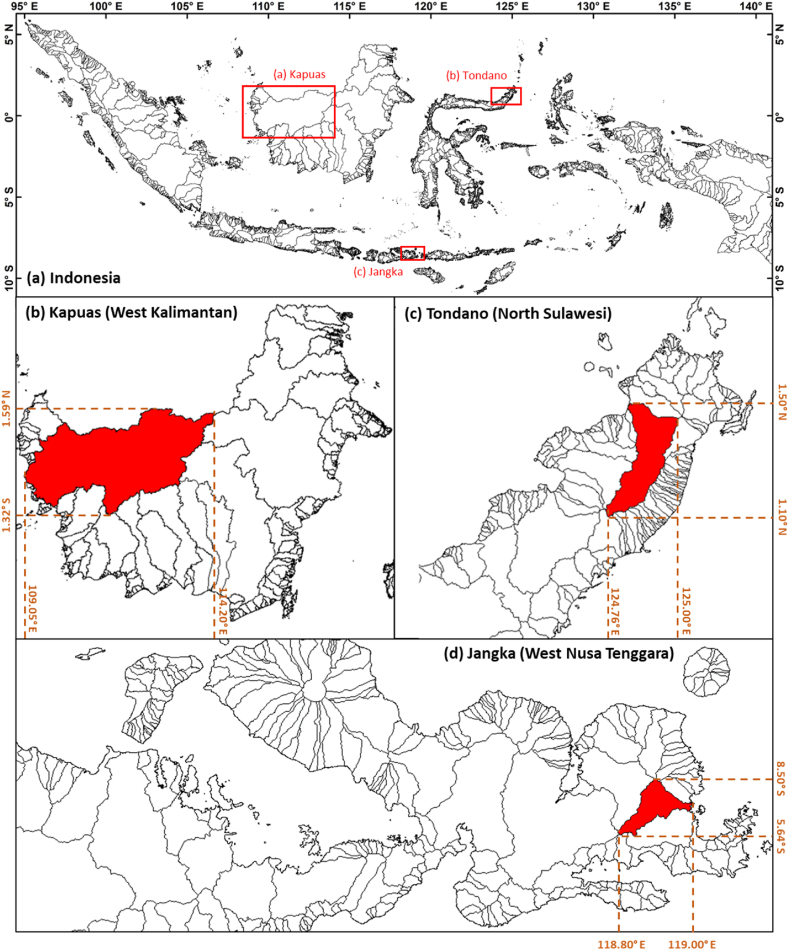
Research locations, a) Indonesia, b) Kapuas Watershed in West Kalimantan, c) Tondano Watershed in North Sulawesi, d) Jangka Watershed in West Nusa Tenggara.

**Fig. 2:**
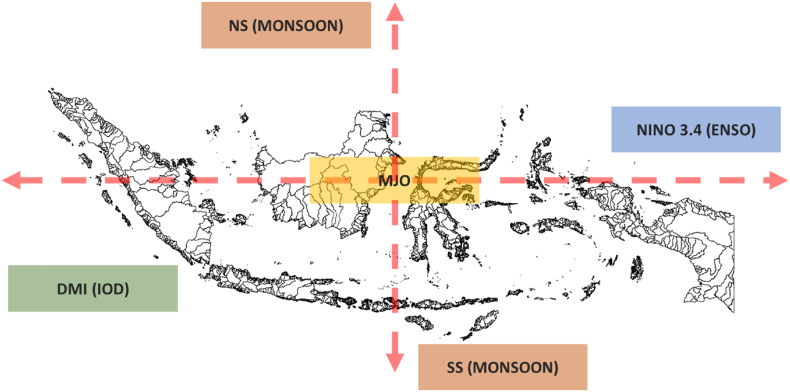
Illustration of atmospheric index data used in this study.

Pontianak, Manado, and Bima areas were selected as the case study locations, with their respective watersheds. The three cities are located sparsely across Indonesia, with different geographical and demographic characteristics. Pontianak is a city located at the equator line and on a relatively nonvolcanic island. The terrain is relatively flat without mountains but with a large area of rainforest in the headwater. Kapuas River crosses the city. In Pontianak, despite abundant water from the Kapuas River, people use rainwater to fulfill their daily needs. The second city, Manado, is located around the northern end of Indonesia, close to the Philippines. The city is located with terrains created by volcanic activities, thus dominated by steep terrain and narrow beaches. Manado people generally use surface water and shallow groundwater. A municipal waterworks network (serving about 30% of the area) also draws water from the river. In Bima, daily water use comes from springs and shallow groundwater. However, the conditions depend on the season, whereas in the dry season, the water flow tends to decrease [37]. The three respective watersheds are Manado's Tondano, Pontianak's Kapuas, and Bima's Jangka (Fig. 1). The three watersheds represent the diversity of watersheds in IMC. The Tondano watershed is in the northeastern region of IMC and the area directly adjacent to the Pacific Ocean. The Kapuas watershed is close to the equator and borders the IMC's inner sea. In contrast, the Jangka Watershed represents the southeastern area of IMC directly adjacent to the Indian Ocean. The relationship of hydrological-meteorological processes at the three locations may differ and depend on each land-sea-air process in the area (see Fig. 2).

### 2.2 Hydrometeorology data & index

Rainfall data from the Global Satellite Mapping of Precipitation (GSMaP) satellite product is used to calculate the drought index value in the 2001 to 2019 range. GSMaP rainfall data have been corrected with field station data [[38], [39], [40]]. GSMaP data was chosen because of the limited rain data from field stations, both spatially and temporally. Rainfall data from the satellite was extracted proportionally to each area of the Kapuas, Tondano, and Jangka watersheds. This is done because the GSMaP grid is 0.1° × 0.1° while the three watersheds analyzed have a larger area than the grid, so one watershed is represented by a set of temporal rainfall data. Streamflow data were obtained from the stream gauges built by the Ministry of Public Works and Housing (PUPR) of the Republic of Indonesia. There is a total of 2,152 stream gauges that continuously measure and record streamflow data all over Indonesia since the 1970s (https://data.pu.go.id/dataset/master-data-aset-infrastruktur-pos-duga-air/resource/54d1721f-5338-4989-94d8-06576848028a). This study uses data which can represent streamflow at each focused watershed location. The selected streamflow data is at the same time interval as the rainfall. Although some minor parts of the obtained data show missing values in several timeframes, it is still possible to proceed and produce an analysis. In this research, streamflow data represent the amount of surface water supply with the assumption that other parameter such as run off, infiltration, and surface evaporation are not considered.

ENSO, IOD, MJO, and Monsoon are the global atmospheric phenomena parameters included in this research. Each of them represented the atmospheric event from different circulation surrounding IMC. Indexes were used to indicate when events occurred. The ENSO represents the phenomenon of the ocean-atmosphere interaction that affects IMC from the east, which is related to the Pacific Ocean circulation [41]. The ENSO was represented by the Nino 3.4 index, which shows the occurrence of El Nino (positive value) and La Nina Period (negative value). El Nino is often associated with long dry phenomena caused by lack of rainfall leading to a vigorous drought intensity for some locations. Meanwhile, the presence of La Nina is often associated with a long rainy season and extreme rains that lead to an excess of streamflow [34,42,43]. The IOD represents ocean-atmosphere interaction that affects IMC from the west, related to the Indian Ocean circulation [17]. The DMI represents the IOD event, in which the positive (negative) value indicates the occurrence of positive (negative) IOD. This circulation is similar to the ENSO, where the IOD Positive (negative) is often associated with the increasing (decreasing) rainfall intensity. El Nino and IOD Positive combination would lead to a stronger dry period. Meanwhile, the combination of La Nina and IOD Negative would lead to a stronger wet period. Other than that, they could cancel each other out.

MJO represents an atmospheric phenomenon in the equatorial area with a *trans*-monthly event in 30–60 days [1,44]. Since two regions separated the study area's locations, two indexes are used to represent the MJO occurrence in every location. MJO100E represents the MJO event for the Kapuas watershed, and MJO120E is used for the remaining locations. The presence of MJO is related to the rainfall frequency and duration increase over the equatorial area. The occurrence of MJO could extend the wet period generated by La Nina and/or IOD Positive. Otherwise, it could decrease the dry anomalies generated by El Nino and/or IOD Positive. The Monsoon events were represented with two indexes: the NS index signifying the Monsoon seasonal wind movement from the north of IMC, and the SS index signifying the same seasonal wind from the south. Each Monsoon index has a positive (negative) value that indicates the increase (decrease) value of rainfall related to the season [43,44]. The presence of the Monsoons could vary dry and wet patterns generated by the ENSO, IOD, and/or MJO through the seasonal period (December-January-February; DJF, March-April-May; MAM, June-July-August; JJA, September-October-November; SON) (see Table 1).

### 2.3 Methods

The SPI (also called the drought index) was calculated to identify rainfall anomalies related to global atmospheric circulation through IMC. The calculations were carried out using the spi_sl_6.exe software (https://drought.unl.edu/monitoring/SPI/SPIProgram.aspx). This software adapts the standardized precipitation index calculation performed by Ref. [28]. Monthly rainfall data with a minimum interval of 20–30 years is prepared for each study location. Each data set is adjusted with a gamma distribution function to determine the probability associated with historical rainfall. Then, the probability value is converted into a rainfall deviation value with an average of zero in the normal distribution using normal inverse estimation. In this normal distribution, the deviation value can indicate the presence of dry (negative deviation) or wet (positive deviation) periods in a specified time (Table 2). The SPI is expressed in different timescales, ideally ranging from 1, 3, 6, 12, to 24 months [28,45,46].

**Table 1:** Atmospheric index data and sources.

No	Data	Information	Source	Duration
1	Cold Surge (CS) and Southerly Surge (SS) Indexes	Wind	http://www.ecmwf.int/	2001–2019
2	Madden-Julian Oscillation (MJO) Index	Wind	https://www.cpc.ncep.noaa.gov/products/precip/CWlink/MJO/	2001–2019
3	Global Satellite Mapping of Precipitation (GSMaP_MVK)	Rainfall	https://sharaku.eorc.jaxa.jp/GSMaP	2001–2019
4	Indian Ocean Dipole (IOD) Index	Sea Surface Temperature	http://www.bom.gov.au/climate/enso/indices.shtml?bookmark=iod	2001–2019
5	El Nino– Southern Oscillation (ENSO)	Sea Surface Temperature	https://www.cpc.ncep.noaa.gov/data/indices/ersst5.nino.mth.91-20.ascii	2001–2019

**Table 2:** SPI Index representation of the wet and dry period.

SPI Index	Representation
2.0 and more	Extremely wet
1.5 to 1.99	Severely wet
1.0 to 1.49	Moderately wet
−0.99 to 0.99	Near Normal
−1.0 to 1.49	Moderately dry
−1.5 to 1.99	Severely dry
−2.0 and less	Extremely dry

The recommendations provided by [27] were to use the 1-month SPI index and 3-month SPI index to identify meteorological droughts and the 6-month SPI index to identify hydrological droughts. SPI compares the rainfall data between selected intervals to all the historical data. The 1-month SPI (SPI1) displays the percentage of normal precipitation for the 30 days. The 3-month SPI (SPI3) compares the precipitation over three months. In other words, SPI3 in February compares the December-January-February total precipitation in that particular year with the December-January-February totals of all the historical precipitation data. The 6-month SPI (SPI6) has similar terms as SPI3 but with the interval data of 6 months [27,28,45,46]. As the SPI value of each location was obtained, the three sets of data could be analyzed: SPI (1-month, 3-month, and 6-month), atmospheric indexes, and streamflow data. The results are used to evaluate whether the watershed's streamflow is variating with the global atmospheric event using statistical significance assessment.

## 3 Results

### 3.1 SPI characteristic based on atmospheric phenomena

**Fig. 3:**
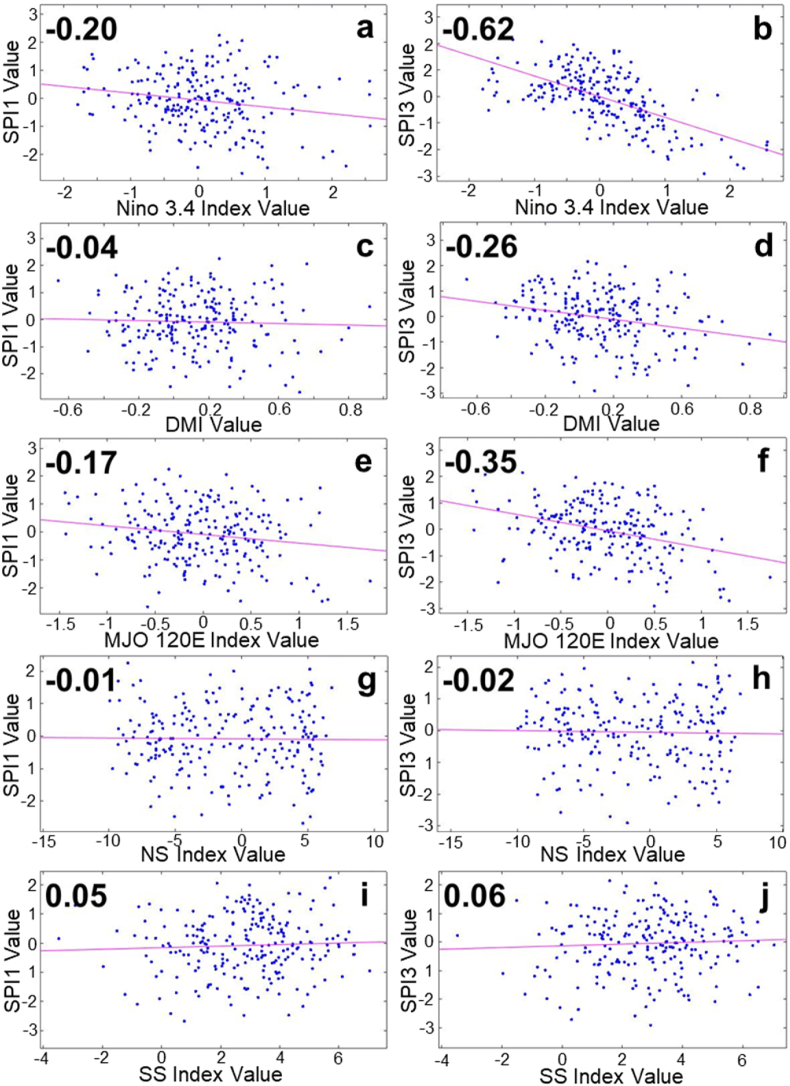
SPI correlation on atmospheric indexes in Tondano watershed. (a) SPI1-Nino 3.4, (b) SPI3-Nino 3.4, (c) SPI1-DMI, (d) SPI3-DMI, (e) SPI1-MJO120E, (f) SPI3-MJO120E, (g) SPI1-NS, (f) SPI3-NS, (h) SPI1-SS, and (j) SPI3-SS.

Atmospheric phenomena are observed for their effect on the drought index at different time intervals, 1-monthly (SPI1) and 3-monthly (SPI3). In the Tondano watershed, the incidence of ENSO and MJO is negatively correlated with a not-too-large number (−0.20 and −0.17) with respect to SPI1 (Fig. 3a and e). Meanwhile, another atmospheric event appears less correlated (Fig. 3c, g, 3i). At the 3-month time interval (SPI3), atmospheric events correlate more with the drought index. ENSO dominance over SPI3 with a negative correlation of −0.62 (Fig. 3b), followed by MJO, which has a negative correlation of −0.35 (Fig. 3f), and DMI, which shows its contribution to the drought index with a negative correlation of −0.26 (Fig. 3d), while the contribution of Monsoon still appears to be the lowest (Fig. 3h and j).

**Fig. 4:**
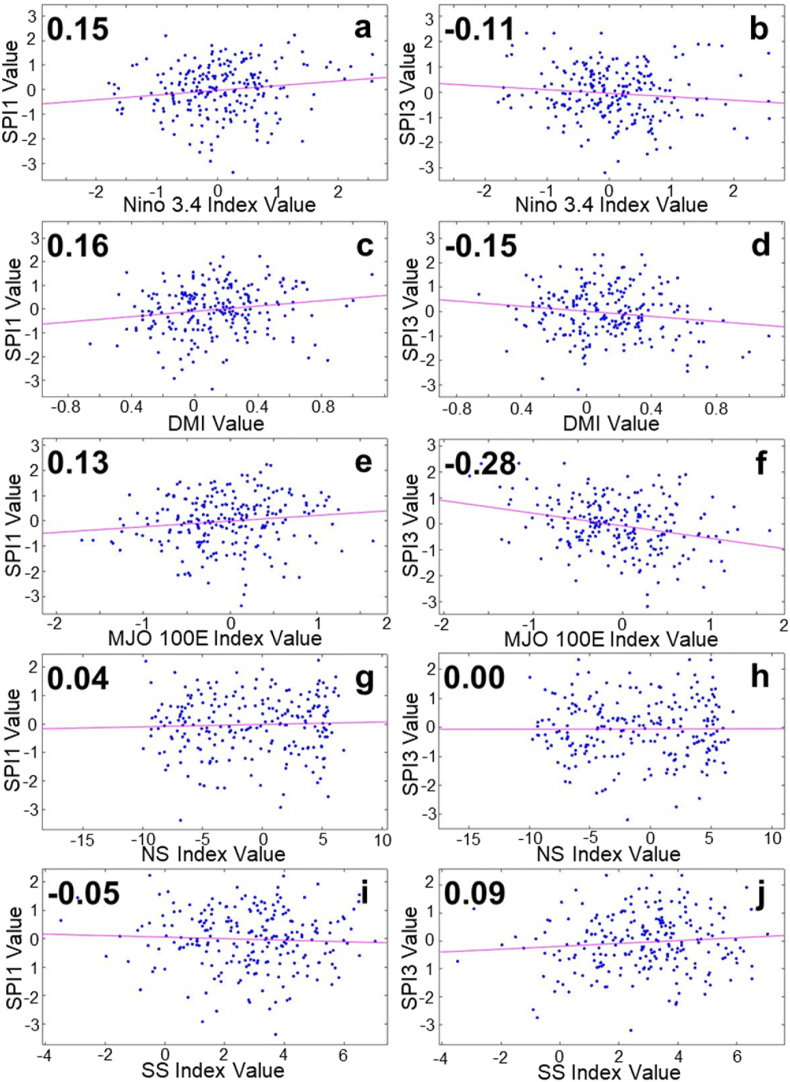
SPI correlation on atmospheric indexes in Kapuas watershed. (a) SPI1-Nino 3.4, (b) SPI3-Nino 3.4, (c) SPI1-DMI, (d) SPI3-DMI, (e) SPI1-MJO120E, (f) SPI3-MJO120E, (g) SPI1-NS, (f) SPI3-NS, (h) SPI1-SS, and (j) SPI3-SS.

In the Kapuas watershed, the contribution of the ENSO, IOD, and MJO phenomena to the 1-month drought index looks more evenly distributed, each with a positive correlation of 0.15, 0.16, and 0.13 (Fig. 4a, c, 4e). On the other hand, the occurrence of Monsoons appears to be less correlated (Fig. 4g and i). At a longer interval (3 months), the correlation of ENSO, IOD, and MJO to the drought index shows interesting changes. Positive correlations that occur at 1-month intervals change to negative correlations at 3-month intervals. This change is seen to be stronger in the MJO-SPI3, which is a negative correlation of −0.28 (Fig. 4f), followed by ENSO and IOD, whose correlation changes to −0.11 and −0.15 values (Fig. 4b and d). Meanwhile, it still has a low correlation value of the SPI3 and the occurrence of the Monsoon (Fig. 4h and j).

**Fig. 5:**
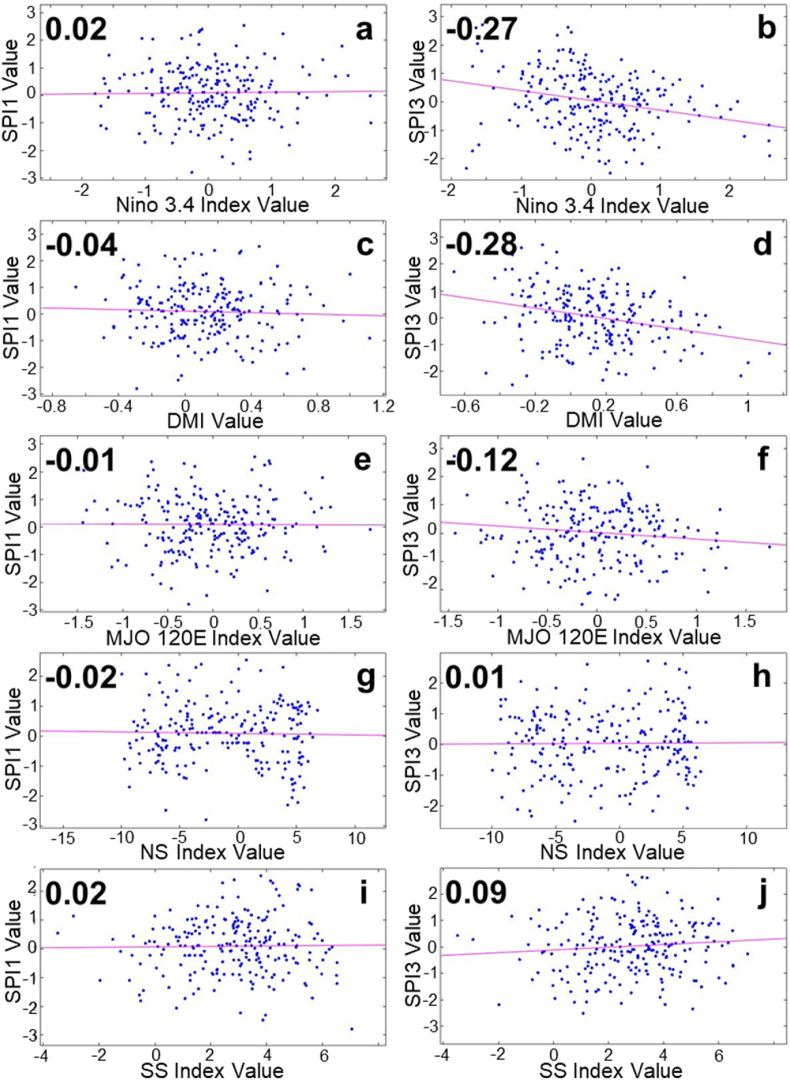
SPI correlation on atmospheric indexes in Jangka watershed. (a) SPI1-Nino 3.4, (b) SPI3-Nino 3.4, (c) SPI1-DMI, (d) SPI3-DMI, (e) SPI1-MJO120E, (f) SPI3-MJO120E, (g) SPI1-NS, (f) SPI3-NS, (h) SPI1-SS, and (j) SPI3-SS.

In contrast to the previous two watersheds, the correlation between atmospheric phenomena and the drought index in the Jangka watershed appears with a low value for any atmospheric events at 1-month intervals (Fig. 5a, c, 5e, 5g, 5i). At 3-month intervals, it is seen that the incidence of ENSO and IOD contributed to the drought index greater than at 1-month intervals, with negative correlations of −0.27 and −0.28, respectively (Fig. 5b and d). The MJO event also began to show its contribution to the drought index with a negative correlation of −0.12 (Fig. 5f). At the same time, Monsoon incidence has a low correlation at monthly and 3-monthly time intervals (Fig. 5h and j).

The negative correlation between Nino 3.4-SPI and DMI-SPI indicates that when El Nino or Positive IOD is active, certain watersheds experience dry periods. In contrast, when La Nina or Negative IOD is active, certain watersheds experience wet periods. The negative correlation between MJO-SPI also indicates that the increasing (decreasing) MJO conditions are associated with dry (wet) periods in certain watersheds. In general, in each watershed, the occurrence of monsoons indicated by the NS and SS index is correlated less significantly, indicating that the dry and wet periods in the three watersheds may vary in minimum on the occurrence of monsoons.

### 3.2 Variability of streamflow during wet and dry period

**Fig. 6:**
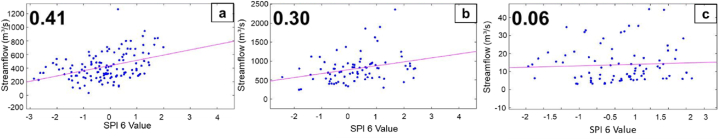
Streamflow-SPI6 correlation in (a) Tondano watershed, (b) Kapuas watershed, and (c) Jangka watershed.

The effect of rainfall anomalies identified through the SPI on surface water flow varies from location to location. A drought index with 6-month intervals was used in this analysis on the recommendations published by WMO. The Tondano, Kapuas, and Jangka streamflows show a positive correlation with SPI6, which means that if the index is positive (wet period), the streamflow tends to increase. On the other hand, if the index is negative (dry period), the streamflow tends to decrease. However, the SPI6 and streamflow are higher in the Tondano and Kapuas watersheds with a positive correlation of 0.41 and 0.30, respectively (Fig. 6a and b), compared to the Jangka watershed (Fig. 6c), where the correlation value is lower (0.06). Fig. 6 shows the correlation analysis result that the Tondano and Kapuas watersheds have a flow that depends on rainfall value, while the Jangka watershed tends to be independent.

## 4 Discussion

The p-value is shown to assess the significance of each correlation value. The 95% confidence interval is chosen, where we could determine that the correlation value is statistically insignificant when the p > 0.05 (the p-value is above 5%). Table 3 shows each SPI-atmospheric index correlation value and its significance level. Monsoon parameters (NS and SS) are the most noticeable, where all the correlation values tend to be statistically insignificant. This means the correlation analysis is not applicable to determine the relationship between SPI and Monsoon occurrence. In a similar way, with the excess of p-value, the correlation between SPI1-atmospheric indexes in the Jangka watershed and the SPI1-DMI in the Tondano watershed tends to be statistically insignificant. Meanwhile, with the little excess of p-value in Kapuas SPI3-Nino3.4 and Jangka SPI3-MJO Index, the correlation value might still be considered.

**Table 3:** The SPI-atmospheric correlation value and *P*-value (significance level) in 95% confidence interval in Tondano, Kapuas, and Jangka watershed.

Watershed Parameters	Statistical Analysis	Atmospheric Parameters				
		Nino 3.4	DMI	MJO Index	NS	SS
Tondano SPI1	Correlation	−0.20	−0.04	−0.17	−0.01	0.05
	*P*-value	0.00	0.54	0.01	0.85	0.45
Tondano SPI3	Correlation	−0.62	−0.26	−0.35	−0.02	0.06
	*P*-value	0.00	0.00	0.00	0.71	0.40
Kapuas SPI1	Correlation	0.15	0.17	0.13*	0.04	−0.05
	*P*-value	0.02	0.01	0.05	0.56	0.45
Kapuas SPI3	Correlation	−0.11	−0.16	−0.28*	0.00	0.09
	*P*-value	0.09	0.02	0.00	0.95	0.15
Jangka SPI1	Correlation	0.02	−0.04	−0.01	−0.02	0.02
	*P*-value	0.79	0.51	0.93	0.72	0.82
Jangka SPI3	Correlation	−0.27	−0.28	−0.12	0.01	0.09
	*P*-value	0.00	0.00	0.06	0.90	0.16

*The SPI-MJO correlation analysis in the Kapuas watershed uses the MJO100E index, while the other use the MJO120E index

**Fig. 7:**
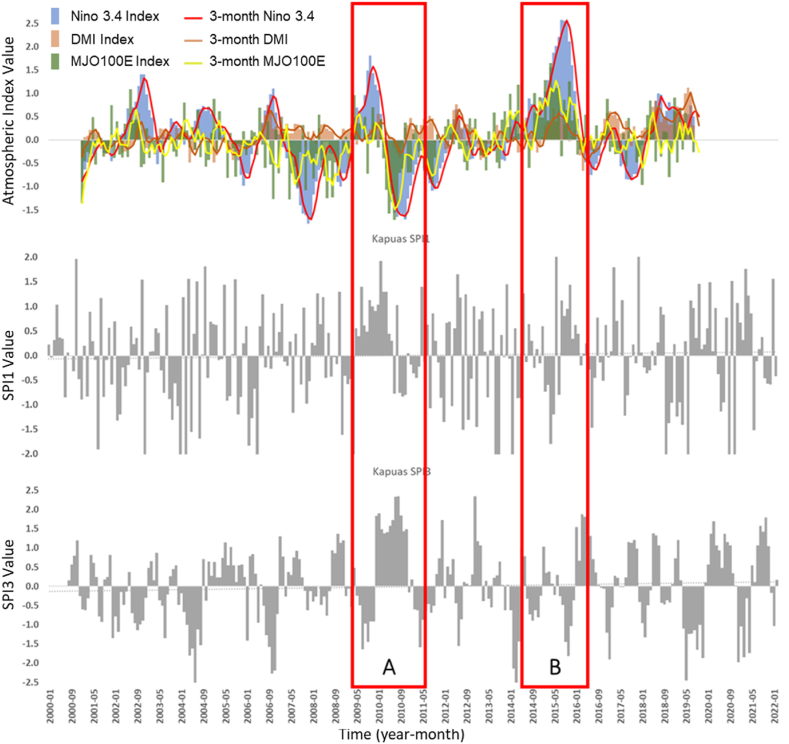
The temporal atmospheric indexes data is juxtaposed with SPI1 and SPI3 data in the Kapuas watershed for the same time span (2000–2022). Nino 3.4 monthly index (blue bars), 3-month average Nino 3.4 (red line), monthly DMI (orange bars), 3-month average DMI (orange line), MJO100E Index (green bars), 3-month MJO100E index (yellow line) 1-monthly SPI (gray bar - middle), and 3-monthly SPI (gray bar - bottom). In the image above, the left axis represents the Nino 3.4, DMI, and MJO100E index values. Red boxes A and B represent the event where MJO strongly cancels the dry periods that strong El Nino and Positive IOD usually generate. (For interpretation of the references to colour in this figure legend, the reader is referred to the Web version of this article.)

Among the three studied watersheds, the Kapuas Watershed provides the most unique SPI-Atmospheric Index correlation value, i.e., a change in the correlation value for SPI1 and SPI3. Meanwhile, for the Tondano watershed and the Jangka Watershed, the correlation between the atmospheric index with SPI1 and SPI3 tends to be similar. However, the correlation value tends to be larger in the SPI3-atmospheric index. The temporal data of the atmospheric index, SPI1, and SPI3, are shown in Fig. 7.

The temporal data (Fig. 7) shows that the SPI1 value (1-month drought index) tends to vary each month and does not directly follow the pattern of atmospheric events, either ENSO, IOD, or MJO. This may indicate that atmospheric phenomena that affect 1-month rainfall in the Kapuas watershed tend to be balanced, no one is more dominant, and their presence cancels each other's effect on monthly rainfall. In contrast to what is present in SPI3 (red boxes A and B in Fig. 7), the wet period in SPI3 occurred when Nino 3.4 showed an intense El Nino event when the dry period should have occurred. That wet period detected by SPI3 may result from MJO compensating for El Nino events. In red box A, it is also shown that when the MJO index is deficient, the strong wet period matches the presence of La Nina on the Nino 3.4 index. The difference in the suitability of the atmospheric index with the temporally seen values of SPI1 and SPI3 may be a factor that causes changes in the correlation value.

**Fig. 8:**
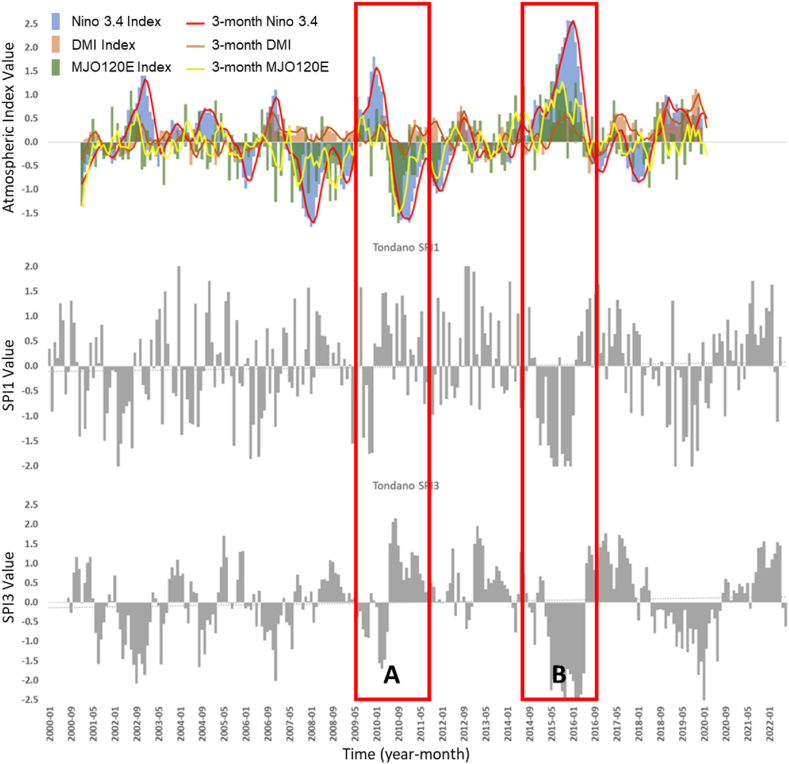
The temporal atmospheric indexes data is juxtaposed with SPI1 and SPI3 data in the Tondano watershed for the same time span (2000–2022). Nino 3.4 monthly index (blue bars), 3-month average Nino 3.4 (red line), monthly DMI (orange bars), 3-month average DMI (orange line), MJO120E Index (green bars), 3-month MJO120E index (yellow line) 1-monthly SPI (gray bar - middle), and 3-monthly SPI (gray bar - bottom). In the image above, the left axis represents the Nino 3.4 and DMI, and MJO120E index value. Red boxes A and B represent the event dry period generated by strong El Nino and Positive IOD. (For interpretation of the references to colour in this figure legend, the reader is referred to the Web version of this article.)

**Fig. 9:**
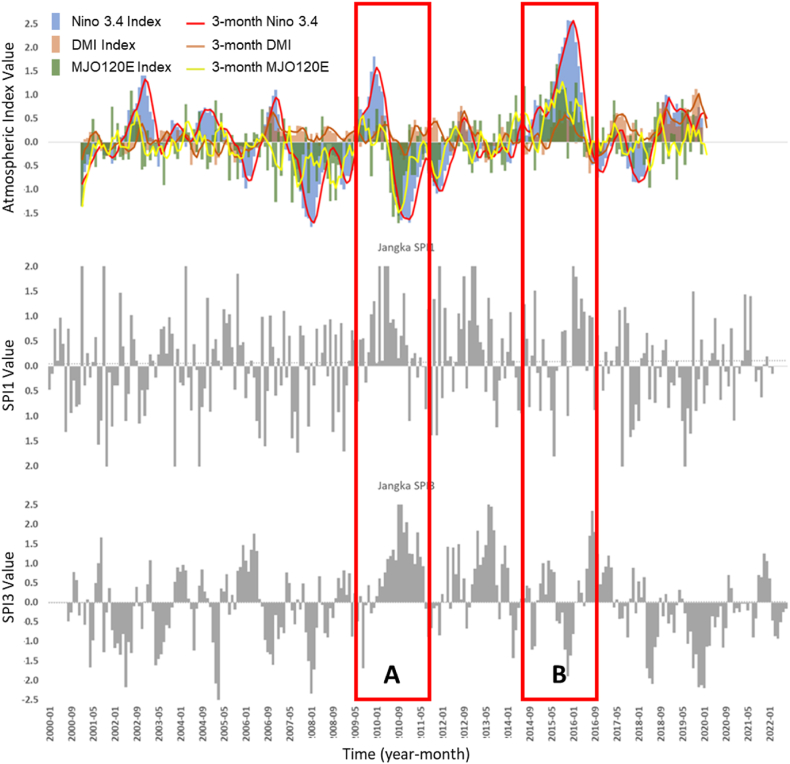
The temporal atmospheric indexes data is juxtaposed with SPI1 and SPI3 data in the Jangka watershed for the same time span (2000–2022). Nino 3.4 monthly index (blue bars), 3-month average Nino 3.4 (red line), monthly DMI (orange bars), 3-month average DMI (orange line), MJO120E Index (green bars), 3-month MJO120E index (yellow line) 1-monthly SPI (gray bar - middle), and 3-monthly SPI (gray bar - bottom). In the image above, the left axis represents the Nino 3.4 and DMI, and MJO120E index value. Red boxes A and B represent the event of the dry period generated by strong El Nino and Positive IOD. (For interpretation of the references to colour in this figure legend, the reader is referred to the Web version of this article.)

The SPI pattern in the Tondano and Jangka watersheds tends to match the temporal pattern seen in the Nino 3.4 index and the DMI (Fig. 8, Fig. 9b). However, the SPI in the Jangka Watershed tends to follow the pattern of the MJO index less, while in the Tondano Watershed, the pattern is still visible (Fig. 8, Fig. 9). Judging from the correlation value, the SPI1 in the Tondano watershed negatively correlates with the Nino 3.4 and MJO indexes, with an undefined relation with DMI. In Jangka Watersheds, each ENSO, IOD, and MJO index's relation with the SPI1 is undefined. The relation shown in the Tondano watershed tends to support the research conducted by Lee [21].

At 3-month intervals (SPI3), the correlation of ENSO and MJO is defined on the SPI3, and the value is stronger (tripled for SPI3-Nino 3.4 and doubled for SPI3-MJO) in the Tondano watershed. In comparison, the correlation value of IOD (SPI3-DMI) is defined at the value of −0.26. At the same time interval, the relation of ENSO and IOD is shown to be noticeable on the SPI3 value in the Jangka Watershed (−0.27 and −0.28, respectively). However, the correlation with the MJO is lower (−0.12). These two watersheds have similar characteristics, which are more highly related to ENSO and IOD events than the Kapuas Watershed. The thing that might connect these two locations is that geographically both are locations that are passed by the Indonesian Trough Flow or ITF (ARLINDO). ITF is an undersurface ocean current that flows from the Pacific to the Indian Ocean through IMC seas. This flow connects the two oceans and flows warm water from the Pacific to the Indian Ocean, which affects the global climate, especially in IMC [41,42]. The Tondano watershed, located close to the ITF entry route (from the Pacific Ocean), and the Jangka watershed, located close to the ITF exit route (to the Indian Ocean), undoubtedly will have different rainfall characteristics and different wet and dry periods. The presence of the ITF and the difference in geographical location can explain why the influence of ENSO and IOD tends to be more on the Tondano and Jangka watersheds than the Kapuas watershed the ITF does not traverse. Table 3 and the temporal data, shown in Fig. 7, Fig. 8, Fig. 9, indicate that ENSO, IOD, MJO, and Monsoon may have different ways of relating to the wet and dry periods. ENSO and IOD tend to relate to the intensity of the wet and dry periods. Meanwhile, the occurrence of MJO tends to modify the wet and dry periods generated by ENSO and IOD. Then, the Monsoon tends to modulate the occurrence of wet and dry periods annually.

Ten sample periods were selected to give a more detailed description of each watershed's wet and dry periods. The events have the most intense high and low SPI3 values provided with Nino 3.4, DMI, and MJO index values. Each index was recalculated by 3-monthly averaging, so the value in each month represents the average index's value of the last three months. The idea was to compare the most intense high and low SPI3 values and atmospheric indexes within the same time span, the three-month average. This could bring more understanding about the links between SPI with the occurrence of atmospheric event as shown in Ref. [22].

The results show different conditions in each study area. In the Tondano, the ten most intense dry periods (severe to extreme) always happen with the activation of El Nino (weak to strong) and rainfall enhancement from MJO (weak to moderate). Only three out of ten most intense wet periods happened with the occurrence of La Nina (weak). The remaining wet periods occurred near the normal Nino 3.4, DMI, and MJO values. In the intense dry period, El Nino impacts stronger to the rainfall anomaly than it could compensate with the enhancement from MJO. The wet period that happens in even normal atmospheric conditions means that the Tondano watershed has a high amount of rainfall all the time. The El Nino occurrence likely impacts the rainfall anomaly rather than La Nina.

In the Jangka watershed, SPI3 correlated more significantly with Nino 3.4 and DMI than other atmospheric indexes, just like the Tondano. However, ENSO dan IOD impacts differently in this area. The ten most intense wet periods (severe to extreme) in the Jangka watershed always happen with the activation of La Nina (weak to strong) and IOD Negative (weak). Although rainfall suppression from MJO occurs for some periods, the wet period is still intense. On the other hand, intense dry periods (severe to extreme) almost always happen in conjunction with the activation of El Nino (weak to strong) and IOD Positive (weak to strong). These findings show that ENSO and IOD anomaly impacts the dry and wet period in the Jangka watershed according to the characteristics of their anomalies.

In the Kapuas watershed, the dominant effect of MJO gave variations to the intense wet and dry periods. In general, the ten most intense dry periods (severe to extreme) occur simultaneously with El Nino and Positive IOD, and the ten most intense wet periods (severe to extreme) occur simultaneously with the presence of La Nina and Negative IOD. Several wet periods (severe) happen when moderate to strong El Nino occur together with weak rainfall enhancement. Occasional rainfall suppression (weak to moderate) occurs in the wet period with La Nina (weak to moderate). Conversely, almost all dry periods occur when El Nino (weak to strong) and positive IOD (weak to strong) are active. However, there was a time when a strong El Nino event was accompanied by moderate rainfall enhancement MJO, so the SPI3 value only reached a severe dry level. Most likely, the rainfall enhancement can compensate for the rainfall anomaly caused by a strong El Nino. From the descriptions, the relationship between atmospheric indexes during the dry and wet periods could be determined in each watershed. ENSO has a broad effect on the occurrence of intense dry and wet periods. The links between ENSO and intense SPI value transpire in several area [[47], [48], [49]].

SPI also connect with the hydrological cycle in watersheds [9,32,33,36,50], therefore the links should be described specifically regarding watershed's characteristic. The Streamflow-SPI correlation value was calculated to define its relation over the watershed. The p-values were also provided to assess each correlation value at the 95% confidence interval. In addition, the amount of streamflow data (N) is also provided. Table 4 shows that the correlation values between streamflow and SPI are statistically significant for Tondano and Kapuas watersheds (0.41 and 0.30, respectively). However, it seems otherwise for the Jangka watershed (0.06).

**Table 4:** Streamflow-SPI correlation value and *P*-value (significance level) at 95% confidence interval in Tondano, Kapuas, and Jangka watershed.

Statistical Analysis	Tondano	Kapuas	Jangka
Correlation	0.41	0.30	0.06
*P*-value	0.00	0.01	0.60
N	126	49	83

The correlation between the streamflow and SPI6 in the Tondano watershed is +0.41; in the Kapuas watershed, it is +0.30; and the Jangka Watershed shows a lower correlation value with +0.06 (note that the value in the Jangka watershed is statistically insignificant). The positive correlation between the two variables means that the streamflow in the watershed tends to increase when the wet period is present. On the other hand, when dry periods are present, the watershed streamflow tends to decrease. Logically, the greater rainfall means the higher streamflow. The inclusion of other variables, such as infiltration, run-off, evapotranspiration, and others, are not yet to be considered.

The Kapuas watershed has the largest area among the three studied watersheds and is included in the ‘huge’ category according to The Watershed Conservation [51]. The Kapuas watershed is included in seven city and district administrative areas, with over 2 million served population by this watershed. In contrast, the Tondano watershed covers four cities and serves around 250–260 thousand population. The Jangka watershed area is much smaller than the other two, only included in one district and serving around 16.790 population. The full data can be seen in Table 5.

**Table 5:** Comparison of catchment area, category, included city, and population served between Tondano, Kapuas, and Jangka watershed.

Criteria	Tondano	Kapuas	Jangka
Area (km^2^) [51]	534	100,284	141
Catchment Category [51]	Tiny	Huge	Tiny
Included City Area [[52], [53], [54]]	4	7	1
Population Served [[52], [53], [54]]	258,674	2,612,416	16,790

From these data, the water use pattern in each watershed may be very different because it is related to the number of people served by the watershed and the main activities of the community in that area. The water use pattern will undoubtedly affect variations in surface water discharge in each watershed. In the Tondano watershed area, the community generally used surface water and shallow groundwater for domestic activities [37]. The main activity of this area is tourism, with the Tondano watershed area being part of the national tourism strategic area [55]. In the Kapuas watershed area, land use is dominated by agricultural and plantation activities [50]. Meanwhile, the Jangka watershed seems to be rarely studied, so it is difficult to know the pattern of surface water use and the dominant community activities there. As a reference, people in the Bima City area generally use deep groundwater to meet their domestic water needs [37,52]. With this study, streamflow characteristics related to ENSO, IOD, and MJO in Tondano and Kapuas watersheds could be determined. For example, when a dry period happens as the result of El Nino or IOD Positive event, it would have a stronger impact on streamflow in Tondano than in the Kapuas. In other conditions, the active MJO around IMC would substantially impact Kapuas streamflow more.

## 5 Conclusions

The SPI value was used to indicate the presence of wet and dry periods at 1-month (SPI1), 3-month (SPI3), and 6-month (SPI6) intervals in the Tondano, Kapuas, and Jangka watersheds. SPI-Atmosphere Indexes correlation analysis result shows that the relation between SPI and Monsoon events could not be determined. The same issues appear in correlation analysis between SPI1-Atmospheric indexes in the Jangka watershed and SPI1-DMI in the Tondano watershed. Other than that, the relation could be determined and have a different characteristic for each studied watershed.

The dry and wet periods in the Tondano watershed are strongly related to the presence of ENSO and MJO events, with correlation values of −0.20 and −0.17 in SPI1; and −0.62 and −0.35 in SPI3. Note that in the SPI3, IOD also has a considerable relation with a value of −0.26. An interesting change in correlation value that appears in the Kapuas watershed is that the positive value on SPI1 turned negative in SPI3. Temporal data shows that the presence of MJO might decline or at least reduce ENSO and/or IOD effect that leads to a dry period in Kapuas Watershed. The MJO also has a more dominant correlation value (−0.28) than the other parameters. Jangka watershed is similar to the Tondano watershed, where the ENSO, IOD, and MJO relation to the SPI3 are considerable. However, it presents a lower correlation value (−0.27, −0.28, and −0.12, respectively) than the Tondano. The ENSO and IOD relate to the intensity of SPI, while the MJO tends to modify the presence of wet and dry periods. Conversely, the Monsoon tends to modulate the occurrence of wet and dry period.

The relation between Streamflow-SPI in Tondano and Kapuas watersheds was determined with the correlation value of 0.41 and 0.30, respectively. With those values, the two watershed's streamflow seems to be considerably dependent on the dry and wet periods. When the wet period is present, the streamflow tends to increase. On the other hand, the dry period would lead to lower streamflow. However, the statistically insignificant (0.06 with a p-value of 0.60) for the Jangka watershed led to undetermined relation between Streamflow-SPI in this watershed. It might be caused by another unidentified atmospheric circulation which should be studied in further research.

With the relation of SPI-atmospheric index and Streamflow-SPI, the variation of streamflow with ENSO, IOD, and MJO over the Indonesia region conjectured. The SPI depends on global atmospheric circulation, and the streamflow depends on SPI, which led to the conclusion that streamflow is related to global atmospheric circulation. In the Tondano watershed, streamflow is considerably related to ENSO, MJO, and IOD. In the Kapuas watershed, streamflow is related considerably to MJO and weakly with IOD and ENSO. This conjecture could be implemented in another area or watershed with similar atmospheric characteristics to Tondano and Kapuas. The correlation among SPI, atmospheric circulation, and streamflow in this research will give strategic information for watershed management since they can prepare for wet and dry seasons based on active atmospheric circulations.

A simple statistical method and analysis have been done in this research to provide basic information about watershed dry and wet period variation in IMC associated with the atmospheric event. Along with the limitation of rainfall and streamflow field station data in each watershed, both temporally and spatially. Historical data on rainfall field measurement and more advanced statistical tools could be implemented if applicable to provide more information in future research.

## Author contribution statement

Fauzan Ikhlas Wira Rohmat: Performed the experiments; Analyzed and interpreted the data; Contributed materials, analysis tools or data; Wrote the paper. Wendi Harjupa: Conceived and designed the experiments; Performed the experiments; Contributed materials, analysis tools or data; Wrote the paper. Dede Rohmat: Conceived and designed the experiments; Contributed materials, analysis tools or data; Wrote the paper; Supervision. Faizal Immaddudin Wira Rohmat: Conceived and designed the experiments; Analyzed and interpreted the data; Contributed materials, analysis tools or data; Wrote the paper.

## Data availability statement

Data will be made available on request.

## Declaration of competing interest

The authors declare that they have no known competing financial interests or personal relationships that could have appeared to influence the work reported in this paper.
